# Age‐related modulation of angiogenesis‐regulating factors in the swine meniscus

**DOI:** 10.1111/jcmm.13218

**Published:** 2017-06-04

**Authors:** Alessia Di Giancamillo, Daniela Deponti, Silvia Modina, Irene Tessaro, Cinzia Domeneghini, Giuseppe Maria Peretti

**Affiliations:** ^1^ Department of Health, Animal Science and Food Safety Università degli Studi di Milano Milan Italy; ^2^ IRCCS Ospedale San Raffaele Milan Italy; ^3^ IRCCS Istituto Ortopedico Galeazzi Milan Italy; ^4^ Department of Biomedical Sciences for Health Università degli Studi di Milano Milan Italy

**Keywords:** Meniscus, pig, endostatin, VEGF, micro‐vasculature, tissue maturation

## Abstract

An in‐depth knowledge of the native meniscus morphology and biomechanics in its different areas is essential to develop an engineered tissue. Meniscus is characterized by a great regional variation in extracellular matrix components and in vascularization. Then, the aim of this work was to characterize the expression of factors involved in angiogenesis in different areas during meniscus maturation in pigs. The menisci were removed from the knee joints of neonatal, young and adult pigs, and they were divided into the inner, intermediate and outer areas. Vascular characterization and meniscal maturation were evaluated by immunohistochemistry and Western blot analysis. In particular, expression of the angiogenic factor Vascular Endothelial Growth Factor (VEGF) and the anti‐angiogenic marker Endostatin (ENDO) was analysed, as well as the vascular endothelial cadherin (Ve‐CAD). In addition, expression of Collagen II (COLL II) and SOX9 was examined, as markers of the fibro‐cartilaginous differentiation. Expression of VEGF and Ve‐CAD had a similar pattern in all animals, with a significant increase from the inner to the outer part of the meniscus. Pooling the zones, expression of both proteins was significantly higher in the neonatal meniscus than in young and adult menisci. Conversely, the young meniscus revealed a significantly higher expression of ENDO compared to the neonatal and adult ones. Analysis of tissue maturation markers showed an increase in COLL II and a decrease in SOX9 expression with age. These preliminary data highlight some of the changes that occur in the swine meniscus during growth, in particular the ensemble of regulatory factors involved in angiogenesis.

## Introduction

Paired menisci are intracapsular semilunar structures aimed at providing congruence of incompatible articular surfaces (namely the femoral condyles and tibial plateau) of the knee joint. Each meniscus is composed of hyaline cartilage, fibrocartilage and connective fibrous tissue, the proportions of which vary in relation with the area (anterior, or posterior horn, central body), the species and the age.

The well‐known meniscus functions of load bearing, shock absorption and joint stability against tension and torsion rely on its specific structural composition, which is characterized by a specialized network of different collagen isoforms, proteoglycans and vascular supply. Various studies have emphasized the complex nature of this structure in different mammals (including humans) and have described the presence of a ‘white–white zone’ in the inner third of the meniscus, characterized by the absence of blood vessels; a ‘red–white zone’ in the middle area, comprising a low number of vessels; and a ‘red–red zone’ in the outer third, composed of a rich vascular network 1, 2, 3, 4. In adult humans, around 70% of each meniscus is avascular or shows a limited vasculature, which results in the reduced possibility of repairing the damaged zones, either spontaneously or by applying sutures 1, 4, 5. This particular distribution of the vascular network in the form of a ‘microvasculature’ is accompanied by a specific biochemical composition of each meniscus, characterized by the prevalence of cartilaginous‐like tissue (mostly collagen II fibres) in the inner part and fibrous‐like tissue (mostly collagen‐I fibres) in the outer part, as exemplified in cows 6 and in pigs 7, 8. This unique collagen composition contributes to the mechanical properties of the meniscus and its healing properties, providing higher resistance to compression, but reduced healing capacity in the avascular zone, and higher resistance to tension and better healing capacity in the vascular zone 1, 2, 3, 4. This specialized structural organization of each meniscus leads to the assumption that its vascular supply and its biochemical composition are simultaneously and reciprocally modulated to adapt the tissues to the different functions that are required throughout growth, until a ‘mature’ phase is reached in adulthood. In fact, it has been previously demonstrated that both the vascular supply and the biochemical composition change with the meniscus maturation 7, 8, 9, 10, 11 in order to conform the meniscal structure to the increasing mechanical stimuli applied to the knee joint. Therefore, it is conceivable that the different messenger molecules regulating vascularity/angiogenesis, and those that regulate the biochemical composition, may be regulated by a common pathway induced by the mechanical stimuli that increase with age. The development and the consequent maintenance of a vascular system are the result of a precise equilibrium between different factors. Specifically, angiogenesis is regulated by a balance of stimulating and inhibiting molecules 12. VEGF is one of the most important angiogenic factors 13. Interestingly, in the knee menisci, high VEGF levels have been found during the healing of meniscus lesions 14, but treatment with VEGF of lesions in the avascular zone of the menisci had no effect on healing in the ovine animal model 15. One of the most important antiangiogenic factors is ENDO, a 20‐kD factor, which was originally studied in murine haemangioendothelioma cells and was shown to counterbalance many VEGF‐induced effects 16, 17, 18. To date, a limited number of publications describe the localization of this factor in human menisci 19 and in human‐cultured fibrochondrocytes 20, 21. In view of this evidence, the present work aims at identifying a spatial–temporal correlation between the expression and localization of angiogenic and anti‐angiogenic factors, along with the expression and localization of molecules involved in the fibro‐chondrogenic commitment of the meniscus, to define how the vascular and the matrix networks evolve during meniscus maturation until adulthood. The study was performed in swine, a largely used animal model of great interest for biomedicine. In addition, different ages were studied in this animal model, bearing in mind that meniscal problems may affect adults (mostly degenerative) as well as young individuals (usually post‐traumatic), and that the morpho‐functional stability of articular connective tissues may vary with different ages.

## Materials and methods

### Study design

The hindlimbs from neonatal (0 day individuals, which had died under the weight of the mother or by natural causes), young (1 month old) and adult (approximately 7 months old) female pigs (Landrace × Large white, average weight 0.7–1, 10–12 and 75–90 kg respectively) were obtained from a local farm (for neonatal and 1‐month‐old individuals) or the slaughterhouse (for adult individuals). To be accurate, we named ‘adult’ the 7 months old pigs in order to make a distinction from the other two younger groups. In reality, related to the pig lifespan, this age should be correlated to a still juvenile animal. The project was approved by the Ethic Committee of the Università degli Studi di Milano, Milan, Italy (OPBA, Protocol number 58/2016). The total number of animals was 12, and the number for each group was 4. The stifle joints were dissected to isolate the tibia and to remove menisci, after having removed ligaments and capsular tissues. According to the similarities observed between the medial and lateral menisci in a previous study from our group 7, and similarly to what other Authors have performed 1, 2, the medial menisci of each joint were analysed. All samples were analysed with both immunohistochemical and Western blot analyses to characterize the vascular network and the fibro‐chondrogenic commitment of the matrix. In addition, the samples were analysed by immunofluorescent analysis to contemporary detect the distribution of angiogenesis‐regulating factors and collagen II fibres (this latter as representative of a mature meniscal matrix) 7.

### Immunohistochemical analyses

Menisci were fixed in 10% (v/v) phosphate‐buffered formalin. For each of the three experimental groups (neonatal, young and adult), four menisci were analysed, each of them further subdivided longitudinally into three portions (the inner, the intermediate and the outer), as above detailed (total specimen nr = 36). All samples were then dehydrated in a graded 50% (v/v), 70% (v/v), 95% (v/v) and 100% (v/v) ethanol series, embedded in paraffin and transversally cut into 4‐μm‐thick sections. The obtained sections were dewaxed and rehydrated and successively treated with 5% H_2_O_2_ in absolute methanol for 10 min., and with normal goat serum (Dakocytomation, Milan, Italy) diluted at 1:20 for 30 min. at room temperature to inhibit non‐specific reactivity.

#### Vascular characterization

A heat‐induced antigen retrieval was applied using a microwave treatment (two times, 5 min., 500 W in 10 mM citrate pH 6.0) before sections were incubated overnight at room temperature in a humid chamber with the following primary polyclonal antisera, all raised in rabbit: (*i*) anti‐VEGF (sc‐152, dilution 1:50; Santa Cruz Biotechnologies, Santa Cruz, CA, USA); (*ii*) anti‐ve‐cadherin (VE‐CAD, abcam, ac33168, dilution 1:200; Abcam, Cambridge, UK); (*iii*) anti‐ENDO (ENDO, code 2161183, dilution 1:200; Millipore, Bedford, MA, USA).

#### Matrix and fibro‐chondrogenic commitment characterization

Mouse monoclonal anti‐collagen type II (COLL II, cod. 7005, dilution 1:400; Chondrex, Redmond, WA, USA) and rabbit polyclonal anti‐SOX9 (abcam, ab264114, 1:100) antibodies were applied overnight at room temperature upon other different sections. The used primary antisera were diluted with a 0.05 M pH 7.4 Tris–HCl saline buffer (TBS: 0.05 M, pH 7.4, 0.55 M NaCl). After the treatment with the primary antibodies has been completed, the antigen–antibody complexes were detected with a peroxidase‐conjugated polymer which carries secondary antibody molecules directed against rabbit immunoglobulins or mouse immunoglobulins (EnVisionTM+; DakoCytomation) applied for 120 min. at room temperature. Peroxidase activity was then detected with diaminobenzidine (DAB; DakoCytomation) as the substrate. Appropriate washing with TBS was performed between each step, and all incubations were carried out in a moist chamber. All sections were finally weakly counterstained with Mayer's haematoxylin, dehydrated and permanently mounted. The specificity tests for the used antibodies were performed by incubating other sections in parallel with: (*i*) TBS instead of the specific primary antibody; and (*ii*) TBS instead of the secondary antibodies. The results of these controls were always negative (*i.e*. staining was abolished). Photomicrographs were taken with an Olympus BX51 microscope (Olympus, Milan, Italy) equipped with a digital camera, and final magnifications were calculated. The observer was not aware of the origin of the sections.

### Protein extraction and Western blot

Medial and lateral meniscal samples were pooled for the Western blotting analysis. The menisci were sectioned longitudinally into the three parts that corresponded to inner, intermediate and the external portions. For each of the three experimental groups (neonatal, young and adult), eight samples were processed, each of them further subdivided longitudinally as previously detailed (total nr = 72). The samples were pulverized for 2 min. at 3000 oscillations/min. in a liquid nitrogen cooled dismembrator (Mikro‐Dismembrator, Sartorius Stedim, Italy). They were then homogenized in a buffer containing 50 mM Tris–HCl, 150 mM NaCl, 0.1% SDS, 0.5% sodium deoxycholate, 1% NP40, pH 7.4, supplemented with protease inhibitor cocktail (Euroclone, Pero (MI), Italy) and centrifuged at 13000 g at 4°C for 10 min. to discard cellular debris. Protein concentration in the extracts was determined using a BCA protein assay (Euroclone). After addition of 0.05% bromophenol blue, 10% glycerol and 2% β‐mercaptoethanol, 50 μg of each sample was boiled and loaded onto 8% SDS–polyacrylamide gels. After electrophoresis, polypeptides were electrophoretically transferred to nitrocellulose filters (Sigma‐Aldrich, Milan, Italy); the membranes were incubated with 5% non‐fat‐milk for 1 hr at room temperature to block the non‐specific sites and then probed for 2 hrs at room temperature using the same antibodies used for immunohistochemistry, as described below:

*Vascular characterization*—anti‐VEGF (1:1000), anti‐VE‐CAD (1:1000) and anti‐ENDO (1:1000);

*Matrix and fibro‐chondrogenic commitment characterization*—anti‐COLL II (1:500) and anti‐SOX9 (1:1000). In addition, a rabbit polyclonal anti‐GAPDH (clone GAPDH‐71.1, 1:2000; Sigma‐Aldrich) as housekeeping protein was used. The membranes were then washed and incubated for 1 hr at room temperature with HRP‐labelled secondary antibodies (1:5000; Bio‐Rad, Hercules, CA, USA). The blots were developed using a chemiluminescent substrate (WESTAR Nova 2011; Cyanagen, Bologna, Italy).

### Statistical analyses

Statistical analysis of the Western blot results (neonatal, young, adult and neonatal *versus* young *versus* adult) was performed using the general linear model of the SAS (version 8.1, Cary Inc., NC, USA). The individual meniscal samples were considered to be the experimental unit of all response variables. The data were presented as least squared means ± S.E.M. Differences between means were considered significant at *P* < 0.05.

## Results

### Immunohistochemical and Western blot analyses

**Figure 1:**
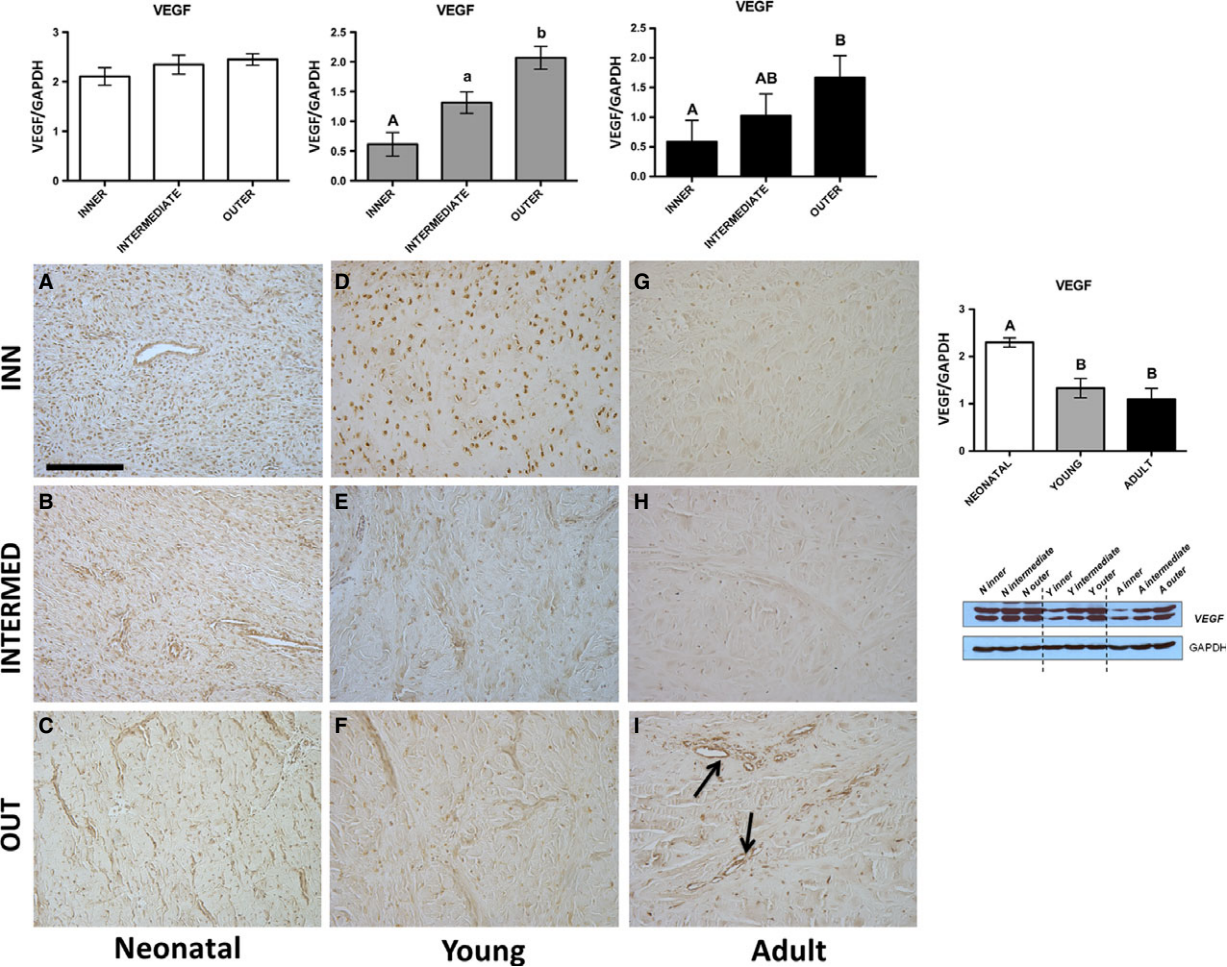
VEGF. Western blot and immunohistochemical appearance in pig neonatal, young and adult menisci. VEGF expression levels obtained by Western blot analysis were measured by densitometry analyses and normalized to GAPDH (housekeeping) levels. Arrows: vessels. All the figures have the same scale bar as located in Figure 3A: 100 μm.

**Figure 2:**
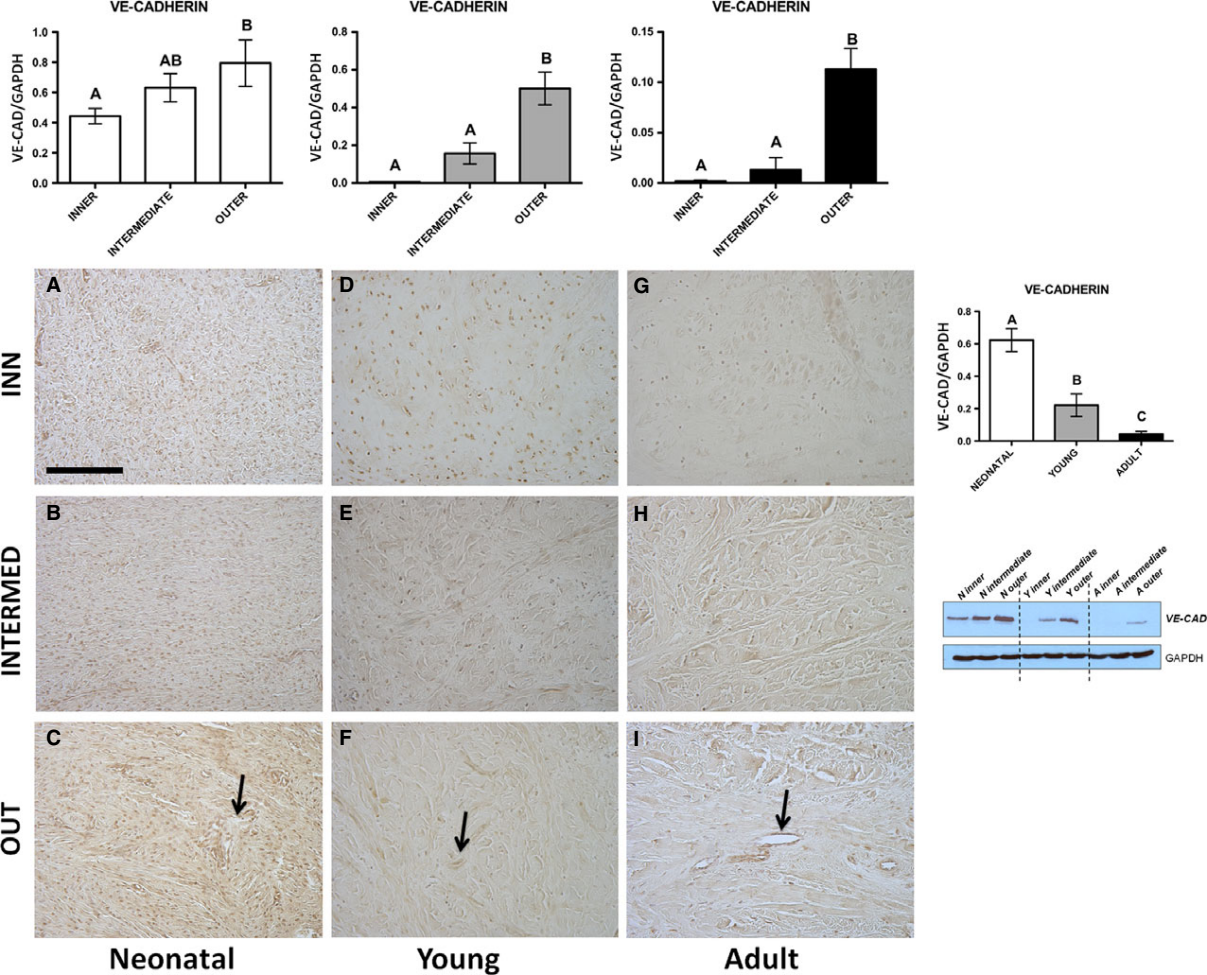
VE‐CADHERIN. Western blot and immunohistochemical appearance in pig neonatal, young and adult menisci. VE‐CADHERIN expression levels obtained by Western blot analysis were measured by densitometry analyses and normalized to GAPDH (housekeeping) levels. Arrows: vessels. All the figures have the same scale bar as located in Figure 1A: 100 μm.

**Figure 3:**
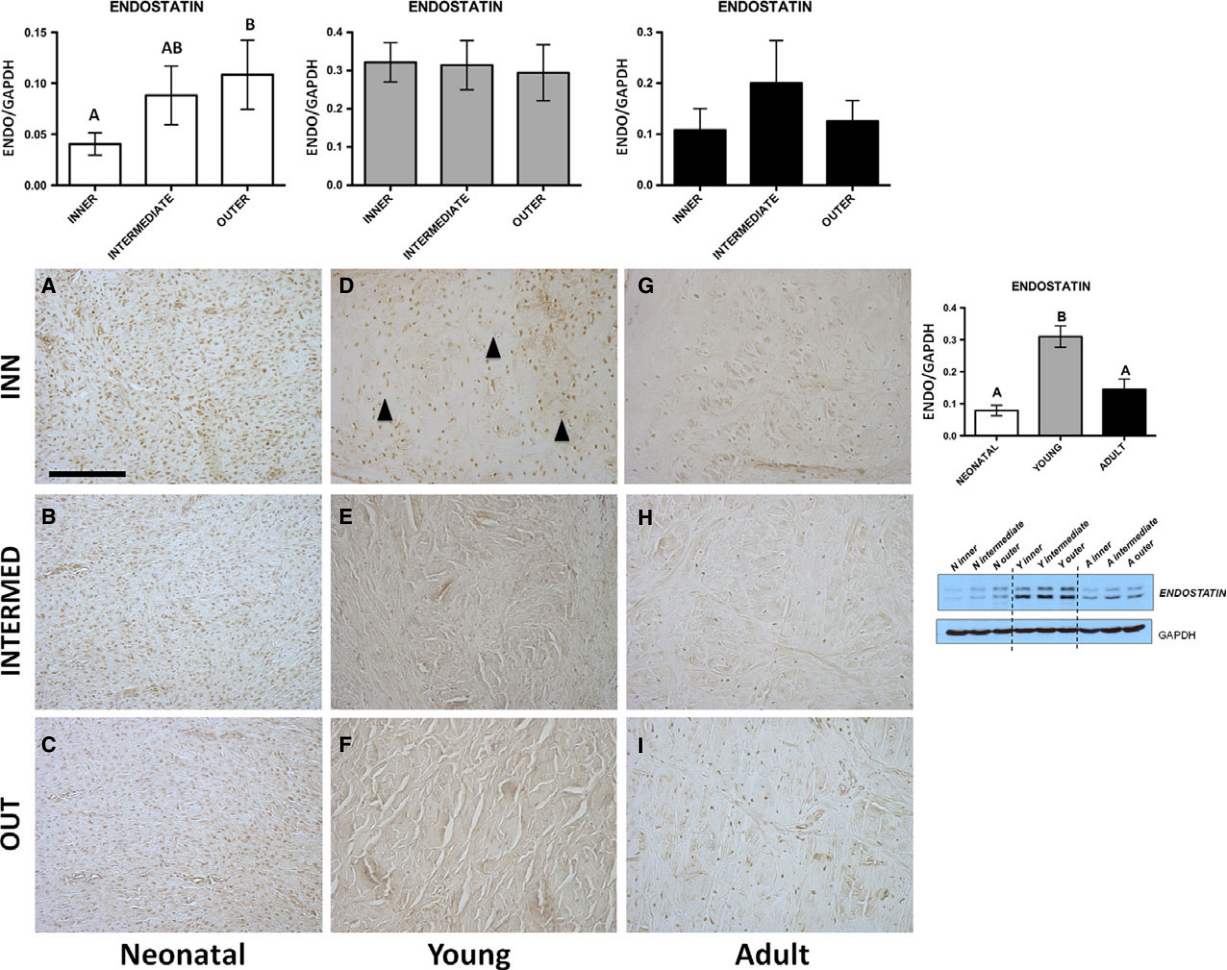
ENDOSTATIN. Western blot and immunohistochemical appearance in pig neonatal, young and adult menisci. ENDOSTATIN expression levels obtained by Western blot analysis were measured by densitometry analyses and normalized to GAPDH (housekeeping) levels. Arrowheads: fibrochondrocytes. All the figures have the same scale bar as located in Figure 2A: 100 μm.

**Figure 4:**
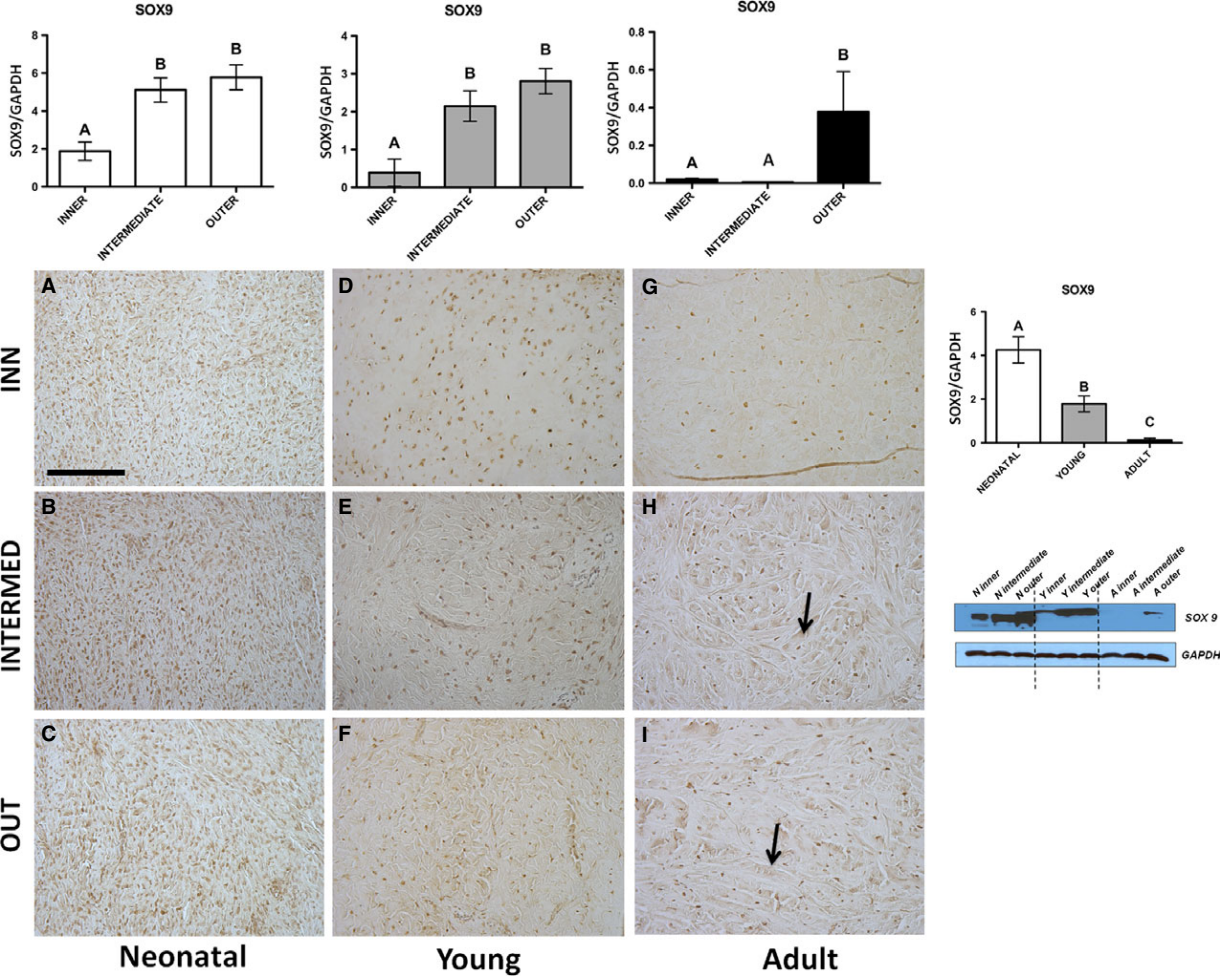
SOX9. Western blot and immunohistochemical appearance in pig neonatal, young and adult menisci. SOX9 expression levels obtained by Western blot analysis were measured by densitometry analyses and normalized to GAPDH (housekeeping) levels. Arrows: fibres. All the figures have the same scale bar as located in (**A**): 100 μm.

**Figure 5:**
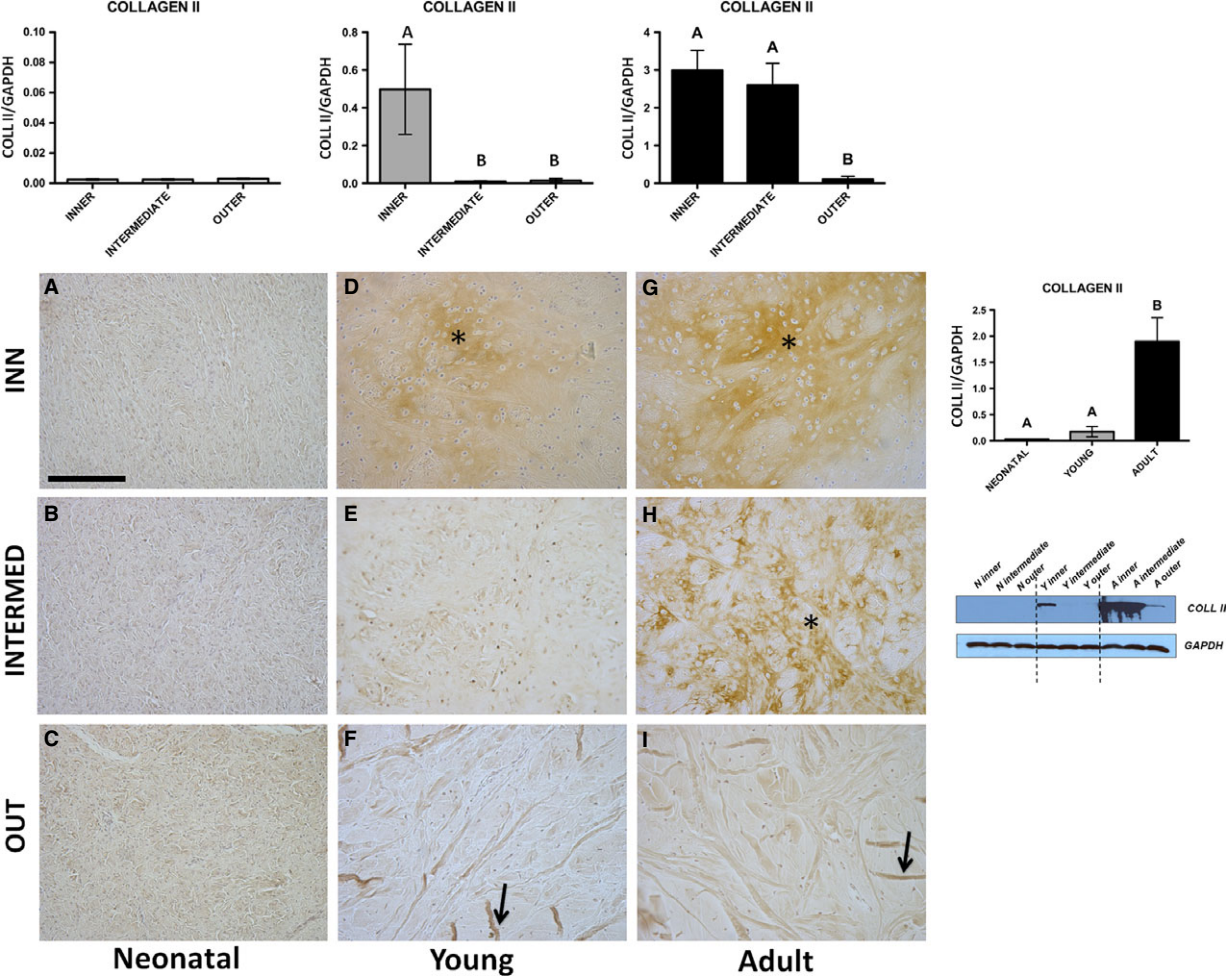
COLL II Western blot and immunohistochemical appearance in pig neonatal, young and adult menisci. COLLAGEN‐2 expression levels obtained by Western blot analysis were measured by densitometry analyses and normalized to GAPDH (housekeeping) levels. Arrows: fibres; asterisk: matrix. All the figures have the same scale bar as located in (**A**) 100 μm.

The results of the immunohistochemical and Western blot analyses are depicted in Figures 1, 2, 3, 4, 5.

#### Vascular characterization

##### VEGF

- Immunohistochemical analysis—An evident immunopositivity was detected in both fibroblasts (Fig. 1A–C) and fibrochondrocytes (Fig. 1D–I), which latter were more abundant especially in the inner zone of the young meniscus (Fig. 1D). In addition, as expected, a VEGF‐immunoreactivity was present in endothelial cells, with a special evidence in the outer zone of the adult (arrows, Fig. 1I).
- Western blot analysis—VEGF revealed no significant differences among the three zones in the neonatal meniscus (Fig. 1, *P* > 0.05), and both the young and the adult samples were characterized by the same trend of significance with increasing VEGF expression from the inner to the outer zone (Fig. 1). Comparing the pooling data, the neonatal meniscus revealed a significantly higher expression of VEGF with respect to the young and the adult ones (Fig. 1, *P* < 0.01).

##### VE‐CADHERIN

- Immunohistochemical analysis—Both fibroblasts (Fig. 2A–C) and fibrochondrocytes (Fig. 2D–I) showed a slight immunopositivity in all three meniscal parts of the examined ages. In addition, an immunoreactivity was detected in the vascular endothelium, more evident in the outer meniscal part of the three examined ages (arrows, Fig. 2C,F,I).
- Western blot analysis—In neonatal, young and adult animals, VE‐CAD revealed the same trend of expression, characterized by a significant increase from the inner to outer part of the meniscus (Fig. 2, *P* < 0.01). As for the VEFG, pooling the three zones, also VE‐CAD, showed a significant higher expression in the neonatal age with respect to young and adult ages (Fig. 2; *P* < 0.01).

##### ENDOSTATIN

- Immunohistochemical analysis—The immunoreactivity was present in fibroblasts of the neonatal age (Fig. 3A–C), as well as in fibrochondrocytes of both the young and the adult (Fig. 3D–I), especially abundant in the meniscal inner part of the young (arrowheads, Fig. 3D).
- Western blot analysis—In neonatal, ENDO showed a trend of expression, characterized by a significant increase from the inner to outer part of the meniscus (Fig. 3, *P* < 0.01), while young and adult animals revealed no quantitative differences among the different meniscal areas (Fig. 3, *P* > 0.05); furthermore, pooling the three zones, the young meniscus revealed a significantly higher expression of ENDO in comparison with neonatal and adult ones (*P* < 0.01).

#### Matrix and fibro‐chondrogenic commitment characterization

##### SOX9

- Immunohistochemical analysis—SOX9 showed an evident immunopositivity in both fibroblasts and fibrochondrocytes in the inner, intermediate and outer parts of all the different stages of maturation of the meniscus. Fibroblasts were especially numerous in the neonatal age (Fig. 4A–C), whereas fibrochondrocytes were present in young and adult ages (Fig. 4D–I), more numerous in the young (Fig. 4D–F). Limited to the latter ages, near to cells fibres are immunopositive too (Fig. 4D–I), in particular at the adult age (arrows, Fig. 4H–I).
- Western blot—In neonatal, young and adult animals, SOX9 revealed the same trend of expression characterized by a significant increase from the inner to outer part of the meniscus (Fig. 4, *P* < 0.01). Pooling the three zones and comparing the three different stages of meniscus maturation, SOX9 showed a significant higher expression in the neonatal when compared to young and adult (Fig. 4; *P* < 0.01).

##### COLL II

- Immunohistochemical analysis—The immunopositivity was very scarce to nearly absent in the three meniscal zones of the neonatal age (Fig. 5A–C), whereas it was present in the young, notably in the meniscal inner zone (asterisk, Fig. 5D). Concerning the adult, the immunopositivity was detected in both the inner and intermediate zones (asterisk, Fig. 5G‐H) especially in the intercellular matrix. A fibrous component was also shown to be immunoreactive, limited to the outer zones of the young and the adult (arrows, Fig. 5F‐I).
- Western blot—COLL II revealed no significant differences in the negligible quantities among the three zones in the neonatal meniscus (Fig. 5, *P* > 0.05), while both the young and the adult samples were characterized by the same trend of significance with higher expression of COLL II in the inner zone (Fig. 5, *P* < 0.01). Comparing the pooling data, the adult meniscus revealed a significantly higher production of COLL II with respect to the young and the neonatal ones (Fig. 5, *P* < 0.01).

### Regulation of vascularization *versus* maturation

**Figure 6:**
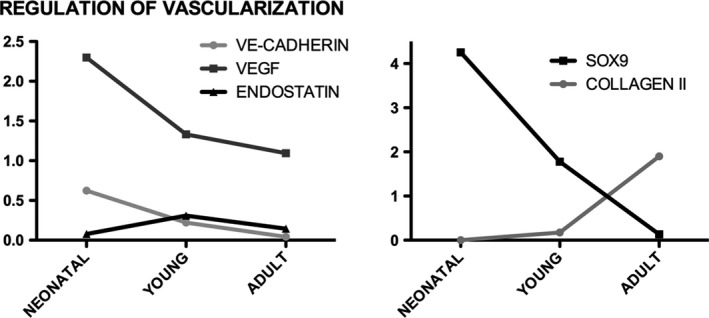
Regulation of vascularization in comparison with meniscus maturation in neonatal, young and adult pigs.

In Figure 6, the regulation of the vascularization is presented, and there is evidence of the same trend of expression for VE‐CAD and VEGF, with a significant decrease from neonatal to adult menisci (*P* < 0.01), while ENDO shows the higher significant expression in the young one (*P* < 0.01). Moreover, meniscus maturation is also presented in order to compare the maturation *versus* the vascularization: SOX‐9 presented a strong decrease from the neonatal towards the adult, while the COLL II revealed a strong increase in expression from the young to the adult.

## Discussion

These data contribute to highlight the changes that occur in the swine meniscus during growth, in particular the ensemble of regulatory factors involved in angiogenesis and the steps leading to the commitment of fibrocartilaginous tissue. Although still preliminary, these are novel results as the vascularization of the meniscus has only been described by a limited number of authors 22, 23, 24. In agreement with these authors, we have observed a reduction in the meniscal vascularization in an age‐dependent manner from neonatal to adult animals. However, our research proposed the characterization of these processes by comparing the modulation exerted by angiogenic *versus* anti‐angiogenic factors during growth. In several other tissues, age‐dependent imbalance between VEGF and ENDO leads to impaired angiogenesis as it has been observed in gastric mucosa 25, besides in prostate 26, in adipose tissue 27 and in retinal epithelium 28, in addition to pathological conditions such as systemic sclerosis 29, 30, intracranial atherosclerosis 31 or acromegaly 32.

As both ENDO and VEGF can be significantly influenced by mechanical factors, and the formation of fibrocartilaginous tissue is a functional adaptation to compressive and tearing forces 10, we studied the modulation and the consequent phenotype characterization of the meniscal tissues from neonatal (with no mechanical stimuli) to adult animals (with mechanical stimuli), with young, growing animals as an intermediate stage.

We observed that VEGF expression decreased from neonatal to adult animals, in accordance with the fact that, in humans, from prenatal development until right after birth, the meniscus is fully vascularized. However, vascularization appears to subside: at 10 years of age, vascularization is present in around 10–30% of the meniscus, and at maturity, the meniscus contains blood vessels in the peripheral 10–25% of the tissue 24. Interestingly, in our study, ENDO had an elevated expression in young animals. Hoberg *et al*. 21 reported that ENDO levels were higher in the internal avascular two‐thirds of the adult meniscus, whereas in the foetal menisci, higher ENDO levels were found in the external area.

These results can be correlated with the application of mechanical stimuli in the meniscal tissue, which increase from neonates to adults; tissue maturation was revealed by observing two distinct areas, namely the outer, vascular region (red‐red zone) and the inner, completely avascular region. Moreover, Pufe *et al*. 33 revealed that mechanical factors influence the expression of ENDO, and that ENDO immunostaining was clearly evident in the avascular zone and gradually reduced in the outer vascularized zone in the tendons. Similarly, Fujii *et al*. 19 observed that Chondromodulin‐I (ChM‐I; anti‐angiogenic factor) significantly decreased endothelial cell proliferation in the inner meniscus. The same authors therefore suggested that ChM‐I may be a key anti‐angiogenic factor for maintaining the absence of vascularity in the inner meniscus. All these data are in accordance with our results. Moreover, we suggest that the passage from the neonatal to the adult condition is modulated by anti‐angiogenic ENDO, which is highly expressed in young animals, according to Pufe *et al*. 5 who considered that ENDO expression was down‐regulated right after birth and could be found in the matrix around the fibrochondrocytes. The spatial and temporal expression of ENDO in the meniscus are important for its development and for the maintenance of avascular zones 5. Other authors indirectly observed the basic role of anti‐angiogenic factors involved in development, for example Foradori *et al*. 34 observed that, in Matrilin‐1‐deficient mice, the angiogenesis during fracture healing was significantly higher in Matrilin‐1−/− mice compared to the wild‐type mice, as demonstrated by elevated expression of angiogenesis markers including PECAM1, VEGFR and VE‐cadherin in that cartilage. When we consider the results of the present study, as a whole, we can observe a clear switch between the expression of SOX9 and COLL II, while the peak level of ENDO corresponded to the intermediate young age. The analysis of maturation markers showed, in fact, an opposite trend, with an increase in COLL II and a decrease in SOX9 expression with age. While SOX9 is an early marker of fibrocartilaginous tissue, COLL II is a marker of adult tissue; this trend in expression is significantly altered at a young age (SOX9 decreases, while COLL II increases 7), thus suggesting that mechanical stimuli play an important role in the vascularization and maturation of the meniscal tissue.

In summary, we have shown that expression of angiogenic and anti‐angiogenic factors in the meniscus depends on age and is directly correlated with the fibrochondrocyte phenotype of the tissue, which is increasingly present with age. A better understanding of the regional variations in the matrix composition and the changes throughout maturation would lead to a deeper knowledge of the events regulating meniscus growth, leading to the development of new strategies for meniscus engineering.

Our results provide new insights regarding the use of specific molecules involved in the regulation of angiogenesis, with the aim of potentially obtaining the basis for developing an engineered meniscus. If properly constructed to include all its specialized functions, an engineered meniscus could then be used when reparation of the meniscus (perhaps in its avascular portion) is necessary, in both human medicine and veterinary medicine. In our view, the importance of vascularization signalling with respect to the healing meniscus tissue should be re‐evaluated in the future.

## Conflict of interest

The authors confirm that there is no conflict of interest.
